# Aberrant B cell responses as drivers of autoantibody generation and epitope diversification in SLE pathogenesis

**DOI:** 10.3389/fimmu.2025.1731285

**Published:** 2026-01-09

**Authors:** Fareeha Tariq, Yathavi Charavanmuttu, Kazi Labiba, Chris Wincup

**Affiliations:** 1 King’s College Hospital, NHS Trust, London, United Kingdom; 2 King’s College London, London, United Kingdom; 3 Guy's, King's and St. Thomas' Medical School (GKT) School of Medical Education, King’s College London, London, United Kingdom

**Keywords:** SLE, epitope spreading, autoreactive B cell, CAR T, rituximab, tertiary lymphoid structures (TLS)

## Abstract

Systemic lupus erythematosus (SLE) is a prototypic systemic autoimmune disease characterised by loss of tolerance, widespread immune dysregulation, and production of diverse autoantibodies (typically directed against nuclear components). A central mechanism underlying this diversification of autoantibodies is epitope spreading, where immune responses directed against primary antigen-derived epitope progressively evolve to recognise additional epitopes, thereby perpetuating autoimmune pathology. Evidence from murine models and longitudinal human studies demonstrate that autoreactive B cells are central to this process, functioning both as antibody producers and antigen-presenting cells that sustain T cell responses. Special pockets within secondary lymphoid organs such as extrafollicular regions and germinal centres are the breeding ground for autoreactive B cell repertoire diversification, while tertiary lymphoid structures (TLS) provide tissue-specific niches for *in situ* diversification, particularly in lupus nephritis and cutaneous lupus. These aberrant B cell responses not only perpetuate autoantibody production but also shape organ-specific pathology. From a therapeutic perspective, rituximab and other anti-CD20 monoclonal antibody therapies deplete circulating B cells but may fail to eliminate plasma cells or fully dismantle TLS, allowing diversification to persist and disease relapses to occur. Early-phase studies of CD19-directed CAR-T therapy has shown potent depletion of naïve and memory B cells with partial reconstitution of predominantly naïve repertoires; however, long-lived plasma cells (LLPCs) and certain pathogenic subsets remain unaffected, leaving the potential for relapse. Dual-target CD19/BCMA CAR-T approaches overcome these limitations by additionally depleting plasma cells, eliminating pre-existing autoreactive clones, and reducing inflammatory pathways, offering a more comprehensive reset of B-cell-driven autoimmunity. Epitope spreading thus represents both a driver of chronic autoimmunity and a therapeutic target, highlighting the need for interventions that precisely disrupt autoreactive B-cell networks while preserving immune function.

## Introduction

**Figure 1:**
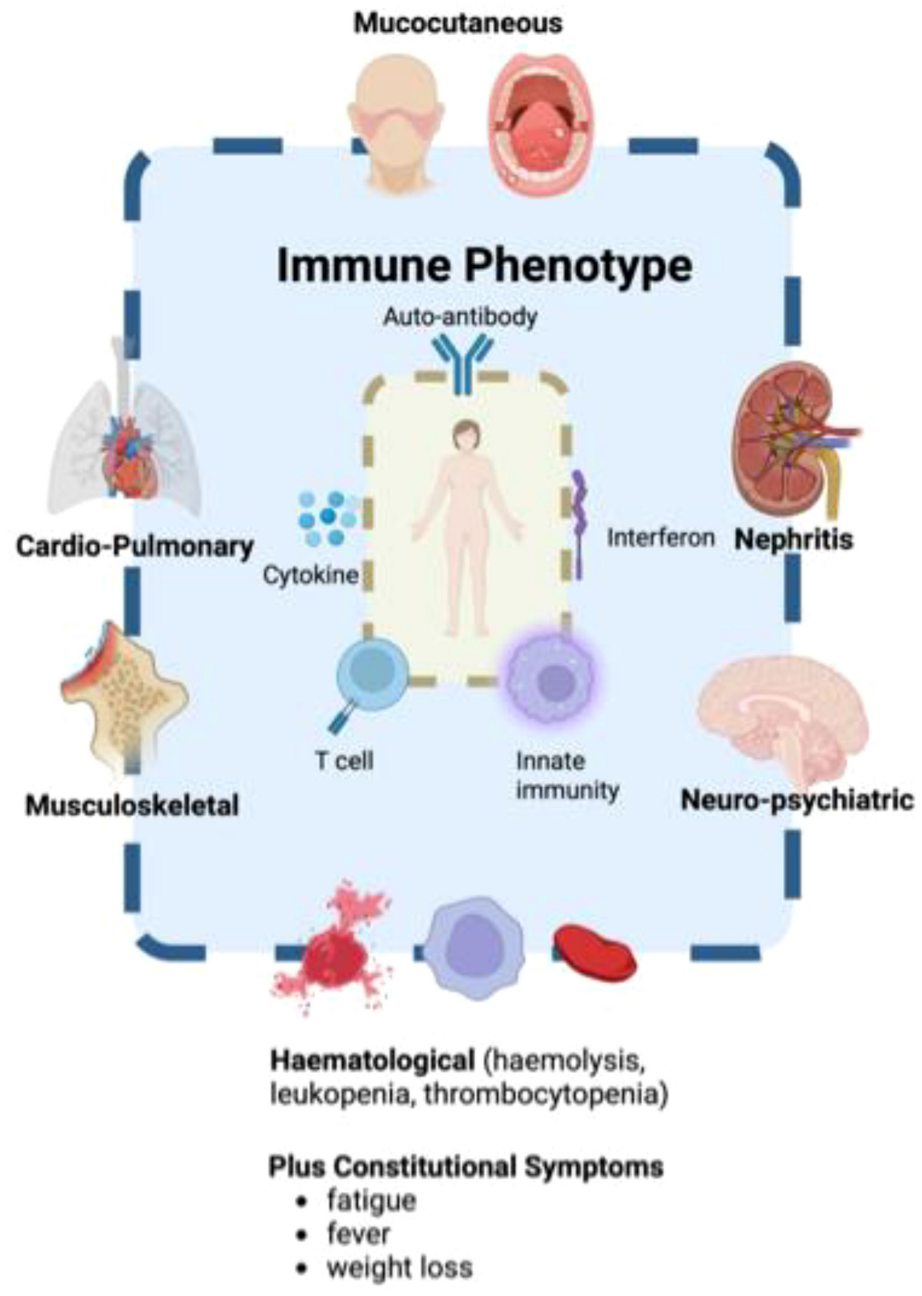
The clinical and immunological heterogeneity of SLE.

Systemic Lupus Erythematosus (SLE) is a chronic autoimmune disease with multisystem involvement (1–3). It is characterised by a loss of immunological self-tolerance, leading to the production of autoantibodies against nuclear antigens, release of cytokines, and systemic inflammation. The clinical presentation of SLE is highly heterogeneous, with multiple organ systems affected including the cardiopulmonary, haematological, mucocutaneous, musculoskeletal, nervous, and renal domains. Common symptoms include oral ulcers, joint pain, and alopecia, whereas arthritis, haematological disorders (such as leukopenia and thrombocytopenia), and malar rash often emerge as the disease progresses (2). This broad array of clinical symptoms and immunological abnormalities are summarised in **Figure 1**.

A diagnosis of SLE relies on the integration of clinical evaluation with immunological testing. The updated 2019 European Alliance of Associations for Rheumatology (EULAR)/American College of Rheumatology (ACR) criteria integrate the sensitivity of the 2012 Systemic Lupus International Collaborating Clinics (SLICC) criteria and the specificity of the 1997 ACR criteria. To first be considered, a positive anti-nuclear antibody (ANA) at titre ≥1:80 is required. This is followed by a weighted scoring system based on clinical (such as fever, leukopenia, seizures and joint involvement) and immunological features (such as antiphospholipid antibodies and low complement levels. A cumulative score of ≥10 supports classification as SLE (4).

Epidemiologically, SLE affects an estimated 3.41 million people worldwide, with incidence varying by geographical region and ethnicity, being highest in North America and lowest in Africa (5). The disease shows a strong female predominance (up to 9:1 compared to men), thought to be attributed to the effects of endogenous oestrogen in enhancing B cell survival and activation, together with genetic and epigenetic factors such as X−chromosome dosage and escape from inactivation (6). SLE is also more common in individuals of African ancestry. Onset typically occurs between 16 and 50 years of age, although reported ranges vary across studies (7). Incidence peaks in women during the reproductive years (20–30 years), whereas in men onset peaks later (50–70 years) (8, 9). The pathogenesis of SLE is multifactorial, involving an interplay of genetic, epigenetic, and environmental triggers such as ultraviolet light, viral infections, and certain drugs (10). These triggers, acting on a genetically susceptible background, promote dysregulated apoptosis and defective clearance of cellular debris, resulting in persistent exposure to nuclear self-antigens (6). These antigens include double-stranded DNA (dsDNA), histones, small nuclear ribonucleoproteins (snRNPs: SmB, SmD, U1-70K, U1-A), and RNA-binding proteins such as Ro (SSA) and La (SSB), which are released in extracellular space due to phagocytopathy. When these self-antigens are recognised by self-reacting or autoreactive B cells, the production of autoantibodies is initiated. These autoantibodies form immune complexes with their corresponding antigens, which circulate in the bloodstream and deposit in small blood vessels and capillaries, most often in high-flow and filtering sites such as kidneys, joints, skin, and the brain (11, 12).

ANA are the most sensitive serological marker present in most patients, and they encompass a wide spectrum of autoantibody specificities. However, distinct autoantibodies within this group are linked to characteristic organ manifestations for instance, anti-dsDNA and anti-C1q with lupus nephritis, anti-Ro/La with cutaneous lesions and congenital heart block, antiphospholipid antibodies with thrombotic events, and anti-NMDA receptor antibodies with neuropsychiatric disease. The wide spectrum of autoantibodies in SLE reflects not only a loss of tolerance but also the progressive broadening of immune responses through epitope spreading. This process, whereby reactivity expands from an initial antigenic target to additional epitopes, underpins the diversification of the autoantibody repertoire and contributes to disease pathology (12–14).

For this review, a targeted literature search was conducted using PubMed to identify relevant studies on autoreactive B cells and epitope spreading in SLE. Search terms included combinations of “epitope spreading”, “autoreactive B cells”, “extrafollicular B cells”, “germinal centre”, “tertiary lymphoid structures”, and “systemic lupus erythematosus”. Additional articles were identified by screening reference lists of key publications.

## Autoantibody repertoire diversification through epitope spreading

Epitope spreading is regarded as critical process in developing systemic autoimmune diseases. It occurs when an initial immune response to an antigen expands over time to target additional antigen sites, activating diverse cells, cytokines, and signalling pathways. Epitope spreading can progress in two ways: (i) intramolecular spreading, where the immune response broadens to target additional epitopes within the same antigen, and (ii) intermolecular spreading, where reactivity extends to epitopes on distinct but physically associated antigens. Although precise molecular mechanisms that drive epitope spreading in SLE are not well understood, this process is central to serological autoantibody diversification (15).

The foundational work on peptide-induced autoimmunity and epitope spreading that recapitulates lupus-like disease comes from animal models, particularly the MRL/lpr mouse strain. In these models, immunisation with synthetic peptides derived from lupus autoantigens has demonstrated that a specific proline-rich peptide sequence, PPPGMRPP, located in the C-terminal of small nuclear ribosomal protein, Sm B/B’, is frequently the initial target of autoimmune response. Responses to this peptide often precede and lead to B cell driven epitope spreading, not only by producing autoantibodies but by presenting processed antigenic peptide to T cells, thereby expanding immune response. This model is further reinforced by findings in New Zealand white rabbits, where immunisation with PPPGMRPP triggers an initial focused response which broadens over time to include multiple epitopes across Sm B/B’ and other spliceosomes proteins such as Sm D, nRNP 70K, and nRNP A and C. Importantly, these rabbits also developed features of clinical lupus. This cross-species consistency highlights that epitope spreading is central to autoantibody diversification and pathogenesis of SLE (15, 16). Similar patterns are also observed in humans, where only a limited set of autoantibodies are detected during the preclinical phase, but the autoantibody repertoire expands markedly by the time of clinical disease onset (17).

Data from longitudinal studies suggest that the overall breadth of autoantibody repertoire may not increase over time, but the dynamics of epitope recognition and intensity of reactivity may change which underscores clinical manifestation. Shifts in epitope recognition within antigenic complexes, most notably the U1-RNP complex, are observed at the time of new organ involvement, consistent with intramolecular epitope spreading (18, 19). In addition, elevated levels of specific autoantibodies, particularly anti-dsDNA and anti-histone H3, show strong correlations with disease activity, while patients with lupus nephritis demonstrate higher overall reactivity, underscoring the clinical significance of these serological changes. These processes are critically shaped by immune cell interactions, with B cells playing a central role in autoantibody generation, antigen presentation, and the amplification of autoreactive responses (20).

## Alternative mechanisms of autoantibody diversification

Although epitope spreading is a likely mechanism of diversification, autoantibody repertoire broadening in SLE may also arise simply through the activation of a range of autoreactive B cells clones in the inflammatory environment. Inflammatory cytokines, such as IFN-α, disrupt multiple B cell tolerance mechanisms, augment the development of autoreactive B cells, and contribute to the significant upregulation of BCR signalling observed in SLE (21–24).

In addition, the already activated autoreactive B cells secrete cytokines such as IL−6 and IL−10, and upregulate survival factors like BAFF, which lower tolerance checkpoints and promote the differentiation of additional autoreactive B cells into plasmablasts and plasma cells (25). Through crosstalk with T follicular helper cells via IL−21 and enhanced responsiveness to IFN−α, these signals create a self−reinforcing loop that sustains and expands the autoreactive B cell pool. This cytokine−driven activation of further autoreactive B cells could contribute to the broadening of the autoantibody repertoire (26).

## Autoreactive B cells as drivers of epitope spreading

Autoreactive B cells arise naturally during B cell development in the bone marrow, where V(D)J recombination in immature B cells generate a highly diverse B cell receptor (BCR) repertoire. A fraction of these BCRs inadvertently recognise self-antigens, posing a risk for autoimmunity. To maintain immune tolerance and prevent autoimmunity, the immune system employs several checkpoints during both central tolerance (in the bone marrow) and peripheral tolerance, such as within the spleen/lymph nodes, also known as secondary lymphoid organs (SLO). Within central tolerance, autoreactive immature B cells may first undergo receptor editing, in which secondary light chance rearrangements provide an opportunity to revise specificity away from self-reactivity (27). If editing fails, highly autoreactive B cells are either eliminated via clonal deletion, through suppression of pro-survival pathways and upregulation of pro-apoptotic proteins. In the periphery, autoreactive B cells that escaped central tolerance mechanisms may persist in a state of clonal anergy, characterised by downregulation of IgM and maintained IgD expression, rendering them functionally unresponsive. These cells can later undergo clonal redemption in germinal centres (GCs), mutating away from self-reactivity while acquiring specificity for foreign antigens (28). Autoreactive B cells are also competitively removed through follicular deletion in SLOs, whereby self-reactive B cells fail to secure sufficient B cell activating factor (BAFF)-mediated survival signals within the follicular niche and are eliminated (29). Although these tolerance mechanisms constrain autoreactivity, accumulating evidence suggests low-level autoreactivity is not merely tolerated but actively required for optimal B cell maturation. A study utilising the Nur77 reporter system identified that clones with minimal self-reactivity are counter-selected, whereas those with modest self-reactivity progress efficiently into mature follicular and marginal zone compartments (30).

Under normal conditions, GCs support the production of high-affinity antibodies through cycles of somatic hypermutation (SHM) and affinity maturation (AM). These processes are orchestrated by follicular dendritic cells (FDCs), which retain and present native antigen to B cells, and T follicular helper cells (Tfh), which provide critical survival and selection signals through CD40–CD40L engagement and IL-21 production. When a fraction of these autoreactive B cells escapes the tolerance checkpoints and continue to receive T cell help, they undergo rounds of SHM and clonal expansion, setting the scene for autoimmunity. These autoreactive clones bind and internalise self-antigen via BCRs and present the antigen-derived peptide on MHC class II molecule to CD4^+^ T helper cells. The presented peptide may differ from the parent epitope recognised by BCR, enabling intermolecular epitope spreading. This cascade activates further T cells specific to newly exposed self-epitopes, which in turn provide help to broader repertoire of autoreactive B cells with distinct self-reactivities. This creates a self-perpetuating torrent of immune activation and epitope spreading (31, 32).

## SLOs support two-step model of epitope spreading in SLE

**Figure 2:**
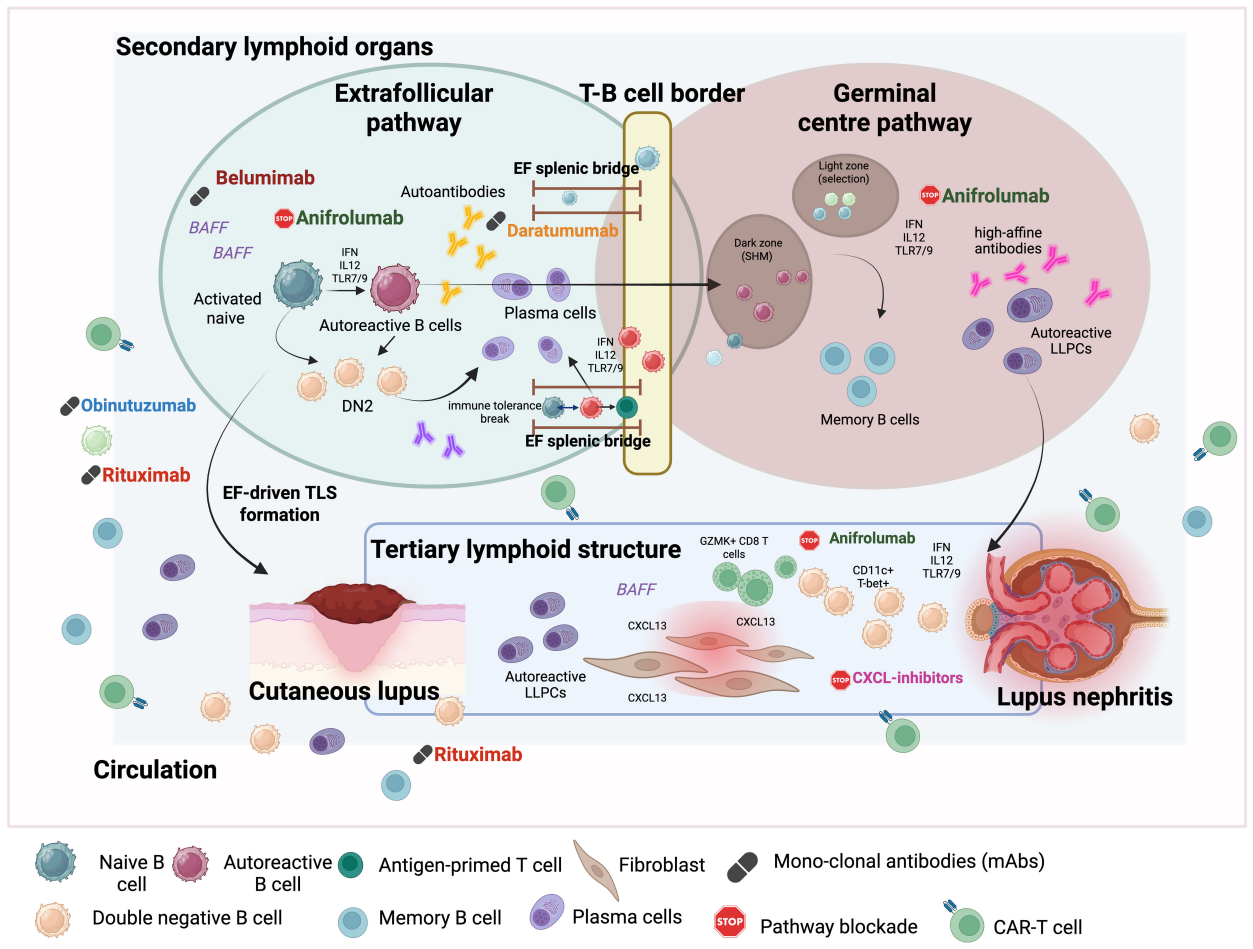
Extrafollicular and germinal centre pathways driving autoreactive B-cell activation and epitope spreading in SLE. Autoreactive B-cell responses in SLE originate primarily within the extrafollicular (EF) splenic bridging channels, where inflammatory cues such as type I interferons, IL-12, BAFF, and nucleic acid-sensing TLR signalling support the initial break in tolerance. In this environment, a single autoreactive B-cell clone can engage antigen-primed T cells, undergo rapid proliferation, and differentiate into short-lived but highly pathogenic plasmablasts. EF expansion generates heterogeneous autoreactive subsets including activated naïve B cells, DN2 (T-bet^+^ IgD^−^CD27^−^CXCR5^−^CD11c^+^/^−^) cells which correlate with disease activity and directly contribute to inter- and intramolecular epitope spreading. Autoreactive B cells that re-enter germinal centres (GCs) undergo somatic hypermutation, affinity maturation, and clonal expansion. GC reactions not only sustain established autoreactivity but can also recruit non-autoreactive naïve B cells into the autoreactive pool, broadening the autoantibody repertoire. These GC-derived cells give rise to long-lived plasma cells and memory B cells that perpetuate systemic autoantibody production. Chronic inflammation and persistent antigen exposure drive autoreactive lymphocytes into target tissues, where they assemble tertiary lymphoid structures (TLS) in organs such as skin and kidney. TLS are sustained by CXCL13^+^ stromal cells, IFN-high myeloid populations, DN2-like B cells, plasmablasts, and GZMK^+^ CD8^+^ T cells, creating a niche that supports local B-cell activation, class switching, plasmablast differentiation, and organ-specific epitope spreading. Therapeutic interventions target distinct points along these pathways: belimumab (BAFF blockade) limits autoreactive B-cell survival; anifrolumab suppresses IFN-driven EF and GC expansion, rituximab depletes circulating CD20^+^ B cells, and CAR-T cells provide deep, CD19-targeted B-cell depletion.

Inside SLO, both extrafollicular (EF) regions and GCs support two-step model of epitope spreading (**Figure 2**). Naïve B cells, which normally reside in B cell follicles, circulate between extrafollicular regions such T-B cell zone, splenic bridges to capture antigen-primed T cells and generate antibodies. These processes are orchestrated by different chemokine signalling such as the CCLR7-CCL19 axis, which guides cells to T-B cell zones, and the CXCR5-CXCL13 axis, which guides cells back to B cell follicles. Once inside follicles, expression of EB12 determines the fate of activated B cells, where upregulation of EB12 directs B cells to EF regions and downregulation commit them to GC designation (33). EF B cells proliferate extensively and rapidly in the interfollicular and bridging channels, differentiating into antibody-secreting plasmablasts. Under healthy condition, these antibodies are of lower affinity than those produced in GCs, however in autoimmune conditions like SLE, EF plasmablasts can nevertheless undergo class switching, somatic hypermutation, and clonal diversification, particularly (34).

Emerging evidence from murine models of SLE (564Igi) suggests that it is the extrafollicular splenic bridges where the initial break in immune tolerance occurs, when a single autoreactive B cell clone interacts with DCs and T cells and subsequently proliferate into autoantibody secreting plasma cells. However, the process of epitope spreading is more complex than mere autoantibody generation and requires expression of dual MHC haplotype by autoreactive B cells to bypass MHC genetic barrier to access help from otherwise haplotype-restricted autoreactive T cells (35, 36). The underlying inflammatory milieu, dominated by type 1 interferons (IFN), interleukin (IL)-12, and nucleic acid-sensing Toll-like receptors (TLR) as well as relaxed tolerance check points in EF regions, further allows expansion of EF autoreactive B cells (**Figure 2**).

These conditions may explain why lupus patients, particularly during disease flares, often exhibit bursts of short-lived but highly pathogenic antibody-secreting cells. Importantly, EF B cells sustain pathogenic processes not only by providing immediate supply of autoantibodies but also acting as potent antigen-presenting cells, sustaining the T-B cell feedback loop via both inter- and intramolecular epitope spreading. Other phenotypes of EF-derived B cells such as activated naïve and double-negative 2 (DN2) cells clonally connect to circulating antibody-secreting cells and directly contribute to serum autoantibodies (37).

Emerging studies link EF DN B-cell subsets with inflammatory signalling and clinical disease activity in SLE. The IFN and TLR pathways in EF environment promote the activation and differentiation of activated naïve B cells into different subsets of DN cells which show significant correlation with disease activity scores in lupus patients. T-bet^+^ DN2 (IgD^−^CD27^−^CXCR5^−^) subsets with variable CD11c expression, are consistently expanded in active disease, and their frequencies correlate with SLEDAI scores. Mechanistic studies have revealed that TLR signalling can promote expression of additional markers on B cells which render them as autoreactive DN2-like cells. For instance, Yasaka et al., 2023 demonstrated that TLR7/9 activation induces expression of phospholipase D4 (PLD4) on surface of B cells. These PLD4^+^ B cells phenotypically resemble autoreactive DN2 B cells and correlate significantly with circulating plasmablast and can generate antinuclear-reactive antibodies, which may contribute to epitope spreading. Genetic variants in TLR7 (rs3853839 G allele) further contribute to DN heterogeneity. A DN subset, DN3 (CD11c^−^CXCR5^−^), which is associated with TLR7 SNP and shows a reduced expression of CD19 has been identified as a strongest biomarker of disease activity in SLE (38–41). The heterogeneity within autoreactive B-cell subsets in the EF region further complicates the cellular and molecular pathways that underlie epitope spreading.

Autoimmune responses mature once autoreactive B cells land in GCs. Murine models of SLE (564Igi) have demonstrated that GCs self-sufficiently drive autoreactivity even from a single autoreactive B cell clone (42). Once this clone triggers TLR-dependent GC activation, it not only undergoes clonal expansion but also recruit other naïve B cells from the wild-type repertoire in the periphery. Within autoreactive GC, these naïve cells acquired autoreactivity through cycles of SHM and AM. The newly formed pathogenic naïve B cells then differentiated into plasma cells to generate autoantibodies against broad range of self-antigens and preserved the autoreactive repertoire by differentiating into memory cells. Once these autoreactive GCs are formed, they behave like normal GC and perpetuate disease pathology by epitope diversification (43). This highlights GCs as potent targets for therapeutic intervention, particularly when therapies that deplete B cells in circulation fail reactive oxygen species (ROS) generation, reflecting heightened demand for adenosine triphosphate (ATP) consumption for rapid clonal expansion and plasmablast differentiation. These metabolic profiles may not only fuel autoantibody production but also promote long-term persistence of autoreactive clones in SLO (44, 45).

## Tertiary lymphoid structures facilitate organ-specific epitope spreading

Emerging from SLO, the movement of autoreactive lymphocytes towards target organs is driven by chronic inflammation and persistent antigen exposure, where they form tertiary lymphoid structure (TLS). In autoimmune disease, TLS elicit organ-level damage and are formed in kidneys, skin, salivary glands, and the joints (46–48). These ectopic lymphoid aggregates serve as local sites of antigen presentation, affinity maturation and autoantibody generation, and thereby facilitate organ-specific epitope spreading. The mechanisms of TLS formation are described in detail elsewhere (49). Akin to SLO, mechanisms of TLS are also driven by chemokines such as CXCL13, CCL19, CCL21, and cytokine IL-7, as they recruit and compartmentalise lymphocytes into B and T cell zones. These zones are maintained by BAFF and lymphotoxin (LTαβ-LTβR) interactions, which rescues autoreactive B cells from deletion and propel them towards AM, class switching, and differentiation into antibody producing plasma and memory cells. The lack of tolerance checkpoints in TLS fuels both intramolecular and intermolecular epitope spreading that drives tissue-specific pathology in SLE (50, 51).

Recent single-cell and spatial transcriptomic studies have refined our understanding of TLS in tissue revealing the cellular and molecular circuits that sustain these pathogenic niches. High-resolution profiling of lupus kidney tissue shows that intrarenal TLS are enriched for T-bet-expressing DN2-like B cells and antibody-secreting cells, with transcriptional program driven by type-I IFN and TLR signalling. BCR repertoire analysis links these DN2 populations clonally to adjacent plasmablasts, providing direct evidence for rapid, *in situ* extrafollicular differentiation and local autoantibody production. These DN2/plasmablast clusters co-localise with IFN-high stromal and myeloid niches, including CXCL13-producing fibroblasts, and receive activation signals from neighbouring pro-inflammatory, cytotoxic GZMK^+^ CD8^+^ T cells to sustain a TLS niche that mechanistically links local extrafollicular B-cell differentiation to organ-specific epitope spreading (52).

In lupus nephritis (LN), inflamed renal tissues are the primary anatomical sites for TLS formation (53), although they may also occur in other tissues such as skin, lungs, or vascular tissues, and are strongly linked with poor prognosis (54–57). The immune cell landscape in LN kidney is enriched for immune cell subpopulations such as APOE^+^ monocytes, GZMK^+^ CD8 T cells, and CD163^+^ dendritic cells (58, 59). The persistence of autoreactive B cells within TLS, together with a network of pathogenic immune subsets could potentially provide an organised niche to support local autoantibody diversification and organ-specific epitope spreading. Resident renal epithelial cells also contribute to these pathogenic niches. Spatial transcriptomics identified VCAM1-expressing proximal tubule (PT_VCAM1) cells in an LN-specific niche in the kidney cortex, where they interact with myofibroblasts and immune cells to promote epithelial-mesenchymal transition and sustain inflammation. PT_VCAM1 cells arise from a failed-repair program regulated by BACH2, and SLE-associated SNPs map to PT_VCAM1-specific cis-regulatory elements, including a BMP2K enhancer containing a BACH2 motif. By organising the niche and engaging immune cells, PT_VCAM1 cells likely support local B-cell activation and extrafollicular differentiation, contributing to TLS-mediated autoantibody production and epitope spreading (60).

Intrarenal B cells from LN biopsies have been shown to produce antibodies not only against classical SLE autoantigens (such as Sm and RNP) but also against locally overexpressed antigens like vimentin, demonstrating that TLS can redirect systemic autoreactive responses toward organ-specific target s. Expression of CXCL13 in macrophages at CXCR5-expressing B cell infiltration sites further helps sustain these autoreactive responses by local autoantibody production. Consistently, patients with TLS in kidneys exhibit elevated serum CXCL13 levels, which underscores it crucial role in tissue level pathogenesis and highlighting it as potential therapeutic target (61). In addition, TLS-positive kidneys often harbour GC-like structures with proliferating autoreactive B cells, which correlate with greater chronicity indices and progression to renal failure in TLS formation in LN. Computational transcriptomic analysis have identified STAT1 and PSMB9 as hub genes strongly linked with TLS formation in LN, highlighting the role of an IFN-driven transcriptional program in sustaining lymphocyte recruitment, antigen presentation, and local immune activation (62).

In cutaneous lupus, TLS sometimes referred to as inducible skin-associated lymphoid structures (iSALT), which may be formed in inflamed skin. Morphologically, these structures initially lack B cells and are generated through recruitment of T effector cells following interactions between dermal dendritic cells and T cells. Autoreactive B cells, that typically reside in SLO, migrate to lesional skin when inflammatory chemokines such as CXCL13, CCL19 and CCL21 are expressed. Unlike SLO, TLS in skin lack full tolerance system to eliminate autoreactive B cells, instead under the influence of rich survival niche composed of BAFF, IL-6, and IL-17, incoming cells persist, differentiate into antibody producing plasma B cells. These structures can also restimulate memory lymphocytes and expand effector responses, making them self-sustaining sites of autoimmunity. Within cutaneous TLS, autoreactive B cells also act as potent APCs through expression of MHC-II, CD40, and CD80/86, facilitating reciprocal T cell activation. It has previously been demonstrated that, following a break in tolerance, B cells expressing dual MHC haplotype can bridge MHC-restricted T cells, therefore facilitate epitope spreading. These processes foster the persistence of autoreactive clones and generation of highly affine antibodies against both skin and systemic autoantigens (63, 64).

## Epitope spreading drives therapy resistance in SLE

Therapies modulating B cell responses in SLE are summarised in **Table 1**. B cell targeting therapies such as rituximab, a chimeric anti-CD20 monoclonal antibody, effectively depletes circulating CD20-expressing B cells, however, it is ineffective in eliminating plasma cells which are devoid of CD20 expression. Relapse in SLE is often attributed to persistence of these plasma cells, to which epitope spreading remains a central mechanism. Rituximab eliminates B cells from circulation as well as TLS to some degree, but it fails to fully dismantle TLS and the stromal scaffold where autoreactive plasma cells and T cells reside (65, 74–76). The vestigial TLS sustains autoreactive T-B cells interaction and internal epitope diversification. Over time, these newly formed B cells reconstitute in the periphery and are recruited to the autoimmune cycle, expanding autoantibody repertoire and directly contributing to relapse. These phenomena have been supported by clinical studies such LUNAR and EXPLORER trials, where rituximab therapy failed to achieve primary clinical endpoint due to reconstitution of peripheral B cells, underscoring the need for repeated dosing or next-generation antibodies (77, 78).

**Table 1:** Summary of B-cell-targeting therapies used in SLE, showing primary targets, effects on specific B-cell subsets, impact on epitope spreading, and key supporting studies.

Therapy	Primary target	Effect on B-Cell Subsets	Impact on Epitope Spreading	Key studies
Rituximab (anti-CD20, type I)	CD20^+^ B cells (naïve, memory, some activated)	Efficient depletion of CD20^+^ naïve and memory B cells Partial depletion of CD20^+^ cells in tissues/TLS and DN2 cells No effect on CD20^−^ plasmablasts or plasma cells	May limit epitope spreading by depleting naïve and memory B cells but incomplete tissue depletion allows autoreactive clones to persist Formation of Anti-drug antibodies (ADA) may act as novel antigens promoting secondary epitope spreading.	Ramwadhdoebe TH, et al., *Rheumatology*, 2019 (65) Faustini et al., Front Immunol, 2022 (66)
Obinutuzumab (anti-CD20, type II)	CD20^+^ B cells with less internalisation	More potent depletion of CD20^+^ B cells and in TLS than rituximab Reduces re-emergence of EF-skewed B-cell subsets	Likely stronger suppression of ES than rituximab due to deeper B cell depletion, though direct evidence is lacking.	Looney et al., Transplant Direct, 2023 (67)
Daratumumab	CD38 surface antigen	Depletion of LLPCs, CD19^low^ CD27^high^ plasmablasts, CD19^+^ B cells	May transiently limit epitope spreading by depleting LLPCs and IFN-driven B-cell activation but effects may be incomplete due to autoantibody regeneration.	Ostendorf et al., NEJM, 2020 (68)
Anifrolumab (anti-IFNAR1)	Type I IFN pathway	Indirectly reduces B-cell subsets (DN2) by blocking type I IFN signalling, which drives autoreactive B-cell responses.	May limit initiation of new autoantibody responses and epitope spreading, but effects are indirect	Dios et al., J Mol Sci, 2025 (69)
CD19 CAR-T	All CD19^+^ B cells (naïve, memory, DN2/DN3, plasmablasts)	Near-complete depletion of naïve, memory, activated, DN2, DN3, and plasmablast subsets Loss of pre-treatment autoreactive memory BCR clonotypes Does not eliminate LLPCs	Strong suppression of new epitope generation; most autoreactive B cells removed; LLPCs remain	Mackensen et al., Nat Med, 2023 (70) Zhou et al., Front Immunol, 2024 (71)
CD19/BCMA dual CAR-T	CD19^+^ B cells, BCMA^+^ plasma cells	Eliminates CD19^+^ B-cell lineages and BCMA^+^ LLPCs Removes autoreactive memory and plasma-cell reservoirs in blood and bone marrow Prevents early re-emergence of EF-skewed B-cell subsets Produces a more complete immune reset than CD19-only CAR-T	Maximal suppression of epitope spreading; prevents re-emergence of EF-skewed B-cell subsets and stops production of new autoantibodies.	Feng et al., Nat Med, 2025 (72) Zhou et al., Front Immunol, 2024 (71)
Belimumab (anti-BAFF)	BAFF survival pathway	Reduction of naïve and transitional B cells Decreases survival of autoreactive naïve and early EF-prone cells Mild effect on memory B cells; minimal effect on plasma cells	Mild-to-moderate reduction of epitope spreading by limiting availability of naïve and EF-prone B cells for new autoantibody generation; memory and plasma-cell compartments allow ongoing autoreactivity and partial spreading.	Huang et al., JCI Insight, 2018 (73)

Epitope spreading can also undermine therapy response through the generation of anti-drug antibodies (ADA), which are reported at high frequencies in SLE patients treated with rituximab and are associated with reduced circulating drug levels and early relapse (79). Because LLPCs and TLS persists after rituximab therapy, autoreactive BCR repertoires continue to diversify toward new antigens, sustaining intramolecular and intermolecular epitope spreading. Concurrently, rituximab itself becomes an iatrogenic target: ADA neutralise the antibody, accelerate its clearance, and further shorten remission intervals. A recent longitudinal immunophenotyping study has demonstrated that although rituximab induces an early reduction in autoreactivity-associated B-cell subsets, most notably the T-bet^+^ DN2 population, this suppression is transient and does not translate into durable remission. Rituximab fails to achieve long-term remission due to emergence of pathogenic B cell subsets with altered phenotype. In particular, the recently described DN3 subset (CD11c^−^CXCR5^−^) which expands early after treatment, suggests that DN3 rather than DN2 subset may represent a rituximab-resistant compartment capable of repopulating the autoimmune niche. Moreover, patients who develop ADA within first 6 months post rituximab therapy exhibit lower overall DN2 B cell frequencies alongside plasmablast expansion. This suggests that early EF-skewed activation, rather than DN2 recovery, drives ADA formation and contributes to therapeutic failure (66). This dual effect of ongoing self-antigen diversification plus therapeutic neutralisation creates a feedback loop that sustains disease activity. Consequently, rituximab treatment may inadvertently fuel further epitope spreading rather than fully suppress it. This explains the mixed outcomes observed in SLE trials compared with conditions like lymphoma, where plasma cells are not central drivers of pathology (80).

The persistence of aberrant B-cell responses remains a key driver of autoantibody diversification and epitope spreading in SLE. Although rituximab provides an effective depletion of circulating CD20^+^ B cells, it does not eliminate LLPCs nor fully dismantle TLS, allowing pathogenic B-cell activity to eventually re-emerge. These challenges have motivated the development of deeper B-cell-targeting strategies. Cellular CD19-directed CAR-T therapy, the type II anti-CD20 monoclonal antibody, Obinutuzumab, and an anti−CD38 monoclonal antibody, Daratumumab, offer the potential for more profound interruption of epitope spreading by more effectively eliminating the autoreactive B-cell compartment.

Daratumumab directly targets long−lived autoreactive plasma cells in the bone marrow and has shown promise in reducing autoantibody titres in refractory SLE (81, 82). By depleting these plasma cell niches, Daratumumab overcomes the limitation of rituximab, thereby offering a more effective strategy to attenuate epitope spreading and disease severity. However, as a type I antibody, Daratumumab undergoes target internalisation, which may limit the durability of depletion compared with type II agents (83). These efficacy constraints mean that while Daratumumab can reduce autoantibody titres, relapse may still occur as autoreactive niches reconstitute over time.

Early clinical data from CD19-directed CAR-T therapy in refractory SLE demonstrate the potential to induce remission accompanied by profound depletion of CD19^+^ naïve and memory B-cell compartments, followed by repopulation with predominantly CD21^+^CD27^-^ naïve B cells during reconstitution. Importantly, the re-emerging B-cell repertoire displays reduced memory B cells and plasmablasts and shifts toward non-class-switched IgM/IgD heavy chains, suggesting a reset of B-cell maturation pathways. However, these are preliminary observations originated from small, well-controlled cohorts and require long-term follow-up. CD19 CAR-T cells do not target long-lived autoreactive plasma B cells, nor there is any evidence of its effect on DN2/3 subsets, which have been associated with ADA in SLE, which leaves open the possibility of future relapse (70, 71). To address these limitations, dual-target CAR-T approaches such as CD19/BCMA CAR-T cells have been developed and recently evaluated in phase I trial in refractory SLE. This approach achieved near-complete depletion of circulating and bone-marrow-resident B cells and plasma cells. Single-cell transcriptomics and BCR-sequencing analyses demonstrated an absence of pre-treatment pathogenic memory B-cell clones during B-cell reconstitution and effective removal of autoreactive B-cell populations. In parallel, key inflammatory pathways such as interferon and BAFF-associated pathway were also reduced, indicating a shift toward a less inflammatory and more regulated immune environment (72).

Within the family of monoclonal antibodies, Obinutuzumab (GA101) has been shown to achieve a more durable B-cell depletion than rituximab. Since GA101 it is a type II agent, it undergoes less CD20 internalisation than type I agent such as rituximab. This ensures sustained target density on B cell surface and enhances depletion efficiency which translates into superior clearance of pathogenic B cells even in secondary lymphoid organs which are reservoirs of TLS (67, 84). Collectively, these data position epitope spreading as a central engine of both disease persistence and therapy resistance in SLE. Partial B-cell depletion leaves plasma cells and TLS scaffolds intact which foster autoantibodies. Mechanistically enhanced anti-CD20 therapy (Obinutuzumab) narrows the gap by sustaining surface target density and improving tissue (including TLS) depletion, while CAR-T therapies offer deeper and broader B-cell and plasmablast depletion, however, their ability to eliminate long-lived autoreactive plasma cells and achieve durable repertoire reset remains under active investigation in early-phase studies.

## Regulatory considerations and safety of CAR-T therapy in SLE

CAR-T cell therapy has shown significant success in inducing remission in various cancers, where its use in autoimmunity remains experimental, which raises regulatory and safety challenges (85). As CAR-T cells are genetically modified, patient-derived cell products, they are classified as advanced therapy medicinal products (ATMPs) and are subject to strict regulatory oversight. In the UK, this oversight is provided by the Medicines and Healthcare products Regulatory Agency (MHRA), which ensures adherence to good manufacturing practice (GMP), quality control, and traceability of all starting materials. This is broadly consistent with the regulatory frameworks appointed globally (86, 87). From a safety perspective, CAR-T therapies are associated with adverse events such as cytokine release syndrome (CRS), immune effector cell-associated neurotoxicity syndrome (ICANS) and cytopenia. These risks can be mitigated through careful patient selection, administration in specialised centres with trained clinical teams, close monitoring of vital signs and laboratory parameters, early intervention with supportive therapies (e.g., tocilizumab or corticosteroids for CRS), and structured long-term follow-up to manage delayed complications (88, 89). For autoimmune diseases such as SLE, careful patient selection, robust preclinical data, and long-term post-treatment monitoring are essential to ensure both efficacy and safety.

## Conclusions

Aberrant B cell responses and the resulting process of epitope spreading are central to the pathogenesis and clinical heterogeneity of SLE. Through extrafollicular activation, germinal centre maturation, and TLS-driven *in situ* responses, autoreactive B cells broaden the autoantibody repertoire, fuelling disease progression and relapse. Current B cell-targeted therapies such as rituximab provide only transient control, as plasma cells and vestigial TLS structures sustain diversification and permit reconstitution of autoreactivity. Emerging CAR T cell therapies offer potential for more comprehensive depletion, reconstituting a naïve B cell repertoire and providing durable immune resetting. Nonetheless, TLS persistence and tissue-resident B cells remain a reservoir for autoreactivity, and long-term immune recovery is variable, posing challenges to sustained disease remission. Future therapeutic strategies should focus on integrating precision targeting of autoreactive clones with modulation of tissue-resident immune niches to achieve long-term disease control without global immunosuppression.
